# Evolution of milk composition, milk fat globule size, and free fatty acids during milking of dairy cows

**DOI:** 10.3168/jdsc.2020-18473

**Published:** 2020-10-29

**Authors:** C. Hurtaud, M. Dutreuil, E. Vanbergue, J. Guinard-Flament, L. Herve, M. Boutinaud

**Affiliations:** 1 PEGASE, INRAE, Institut Agro, 35590, Saint-Gilles, France; 2 Institut de l'Élevage, Monvoisin, 35650 Le Rheu, France

## Abstract

The objective of this study was to measure milk composition (fat, protein, and calcium contents; fatty acid profile), milk fat globule size, and free fatty acid content throughout milking. Composition was measured from milk samples collected every 1 min during morning milking in 2 previously published experiments. Experiments 1 and 2 used 9 and 6 dairy cows, respectively. From the beginning to end of milking in experiments 1 and 2, lactose content decreased (−0.45 percentage units), as did protein content (−0.28 and −0.17 percentage units, respectively). In contrast, fat content increased (+5.66 and +5.57 percentage units, respectively). Milk fat globule size increased (+1.51 and +0.43 µm, respectively), whereas free fatty acid content (measured after 24 h of storage at 4°C) decreased quickly during the first minutes (−0.45 mEq/100 g of fat from time point 1 to time point 4 in experiment 1, and −0.85 mEq/100 g of fat from time point 1 to time point 5 in experiment 2), and then largely stabilized, with a slight tendency to increase toward the end of milking period in experiment 2 (+0.32 mEq/100 g of fat). The evolution of milk fatty acid composition depended on the experiment. From the beginning to the end of milking, the concentration of C16:0 consistently increased (+3.4 wt/wt % in experiment 1 and +3.3 wt/wt % from time point 2 to time point 7 in experiment 2), whereas the C18:1/C16:0 ratio increased during the first minutes of milking and then slightly decreased (−0.050 in experiment 1 and −0.031 from time point 2 to time point 7 in experiment 2). Calcium content decreased in experiment 2 (−58 mg/kg). In conclusion, milk composition changed greatly during milking, suggesting that different mechanisms are involved in synthesis and excretion, depending on the type of milk component.

## Highlights

• Milk composition (fat, calcium) changes during milking
• Milk fat globule size increases during milking
• Lipolysis decreases rapidly during the first minute of milking and then stabilizes

## Graphical Abstract

**Figure:**
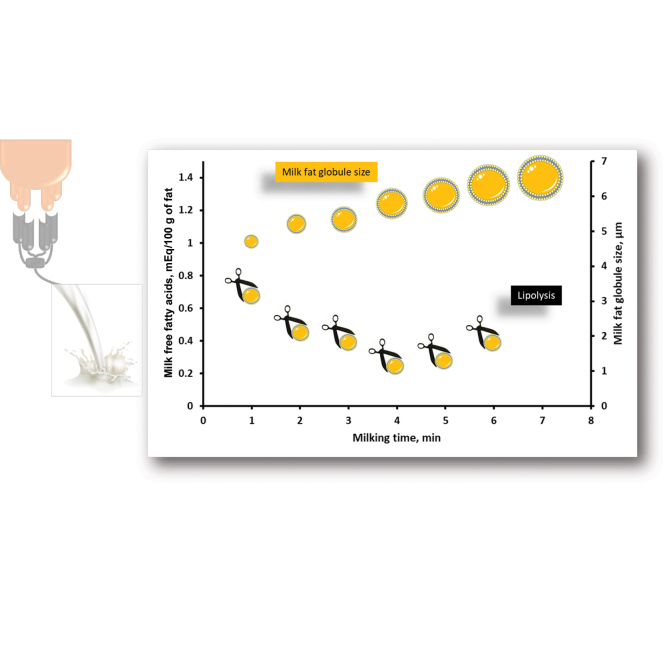
Summary: During milking, the milk fat globule (yellow circle) size increases, whereas the free fatty acids content (indicating lipolysis) (yellow circles with scissors) decreases quickly during the first minutes and then stabilizes.

In dairy cows, milk fat is organized in milk fat globules (**MFG**), which are droplets of triglycerides surrounded and stabilized in the aqueous phase by a 3-layer membrane of proteins and phospholipids that is derived from the outer leaflet of the endoplasmic reticulum and the apical plasma membrane of the cell (Lu et al., 2016). These MFG can be degraded by the enzyme lipoprotein lipase (**LPL**). During this degradation (lipolysis), free fatty acids (**FFA**) are released into the milk. Lipolysis may be responsible for technological and organoleptic defects in dairy products. Evolution of the size (diameter) of MFG and the release of FFA during milking are not well understood (Guinard-Flament et al., 2001), although milk is known to be enriched in fat during milking (Ontsouka et al., 2003; Rico et al., 2014). We studied the evolution of milk composition [fat, protein, lactose, and calcium contents, and fatty acid (**FA**) composition], MFG diameter, and FFA concentration throughout milking, making use of milk samples collected during 2 previously published experiments (Dutreuil et al., 2016; Herve et al., 2017).

Both experiments were conducted at the INRAE experimental farm of Méjusseaume (PEGASE, Le Rheu, France). All procedures used on animals were approved by the local Ethics Committee in Animal Experiment of Rennes (France) in compliance with French regulations (decree no. 2001-464; May 29, 2001; https://www.legifrance.gouv.fr/eli/decret/2001/5/29/AGRG0001697D/jo/texte). In experiment 1 (Dutreuil et al., 2016), 6 mid-lactation Holstein dairy cows (4 primiparous and 2 multiparous) were used. At the beginning of the experiment, their mean lactation stage was 117 ± 22 DIM. They produced 34.3 ± 3.6 kg/d of milk with a mean fat content of 4.0% ± 0.48% and protein content of 3.2% ± 0.21%. The cows were fed corn silage (64.7%), energy concentrate (15%), soybean meal (8%), dehydrated alfalfa (10%), and minerals, vitamins, and urea (2.3%) (Dutreuil et al., 2016) according to INRA (2010) recommendations. In 3 sequential periods, the cows were milked once per day at 0645 h, then twice per day at 0645 and 1745 h, and then twice per day at 0645 and 1045 h (i.e., 24-, 13-, or 20-h milking interval). During one morning milking, milk was sampled once per minute using an inline milk sampler. At each sampling, the quantity of milk produced per minute was recorded. During the last minute of milking, the volume of milk collected in the samplers was too low to perform all analyses; the content of FFA, fat, protein, and lactose could not be determined.

In experiment 2 (Herve et al., 2017), 9 multiparous Holstein dairy cows (lactation 3.1 ± 0.93, 57 ± 5 DIM) were used. At the beginning of the experiment, they produced 40.6 ± 1.36 kg/d of milk with a mean fat content of 3.8% ± 0.09% and protein content of 3.3% ± 0.16%. The cows were fed corn silage (59.0%), energy concentrate (12.7%), soybean meal (14.2%), dehydrated alfalfa (13.1%), 300 g of minerals and vitamins, 200 g of calcium carbonate, and 200 g of sodium bicarbonate according to INRA (2010) recommendations. The cows were milked twice per day (0700 and 1700 h). During one morning milking, milk was sampled once per minute by alternating 2 milking devices. We used a system with a single claw (Westfalia, Hagen, Germany), which was placed at the start of milking. It was a “Y” system, on which it was possible to fix 2 jars and alternatively choose to send the milk to one jar or to the other. Every minute, the jar to which the milk was sent was changed and the milk from the first jar was transferred to a bucket to collect the sample. At each sampling, the quantity of milk produced per minute was recorded. The vacuum was 48 kPa and the pulsation was 60/40.

In both experiments, milk fat, protein, and lactose contents were determined using an infrared dairy analyzer (Milkoscan, Foss Electric, Hillerød, Denmark) according to ISO 9622 (ISO, 2013). Milk FFA concentration was measured, after 24 h of storage at 4°C (t = 24 h; experiment 1) or after 0 or 24 h of storage at 4°C (t = 0 and t = 24 h; experiment 2), in the milk from the morning milking using the copper soap method (Jellema, 1991). In experiment 1, mean MFG diameter was measured in 3 replicates from each cow for each period using a Coulter Multisizer II system (Coulter Electronics Ltd., Luton, UK) fitted with a vacuum suction control unit pierced with a 70-µm-diameter hole. In experiment 2, mean MFG diameter was measured using a Mastersizer 3000 (Malvern Panalytical, Malvern, UK). In both experiments, the De Broukere mean diameter (*d*_4,3_) was calculated as Σ(*N_i_* × *d_i_*^4^)/Σ(*N_i_* × *d_i_*^3^), where *N_i_* = number of MFG with diameter *d_i_*. It reflects the size of particles that constitute the bulk of the sample volume, and it is most sensitive to the presence of large particles in the size distribution. The FA profile was determined from one replicate via lipid extraction from a 0.5-mL sample of milk fat according to Bauchart and Duboisset (1983), using a Varian CP-3800 GC (Varian Inc., Walnut Creek, CA) equipped with an electron ionization detector. The methyl esters of FA were separated on an SP 2560 fused-silica capillary column (100 m × 0.25 mm i.d., Supelco Inc., Bellefonte, PA) under the following temperature program: starting at 90°C for 7 min, increased by 7°C/min to reach 155°C, increased by 3°C/min to reach 235°C, and then held at 235°C for 10 min. In experiment 2, milk calcium content was analyzed by atomic absorption spectrophotometry (Spectra-AA20 Varian, Les Ulis, France; Murthy and Rhea, 1967; Brûlé et al., 1974).

The experimental scheme and milk sampling differed within experiments 1 and 2 due to variability in morning milking durations (6 to 16 min and 7 to 13 min, respectively) and milk yields (15.4 to 28.8 kg and 17.3 to 25.5 kg, respectively). Consequently, data of each experiment were analyzed separately. In both experiments, a 7-point time scale was created for each cow, as described by Herve et al. (2017). The first 3 points corresponded to the first 3 min of milking, respectively, and the last 3 points corresponded to the last 3 min of milking, respectively. The middle point on the scale (point 4) corresponded to the mean value of samples from the intermediate minute.

For both experiments, the factors used to construct the statistical model were cow, time point number, duration of total milking as covariate, and, for experiment 1, period, and milking interval. For experiment 1, the linear model for each variable studied (*Y_ijklm_*) was as follows:

*Y_ijklm_* = *μ* + *V_i_*_(_*_k_*_)_ + *P_j_* + *M_k_* + *N_l_* + *M_k_* × *N_l_* + β(*MD_ijklm_*) + *e_ijklm_*,

where *μ* = overall population mean, *V_i_*_(_*_k_*_)_ = effect of cow *i* having followed *M_k_, P_j_* = effect of period *j, M_k_* = effect of milking interval (13, 20, or 24 h), *N_l_* = effect of time point *l, M_k_* × *N_l_* = interaction between milking interval and time point number, β = regression coefficient and *MD_ijklm_* = duration of milking, and *e_ijklm_* = error associated with each *Y_ijklm_*. For experiment 2, the model was the same without *P_j_, M_k_*, and *M_k_* × *N_l_*.

The data were analyzed by ANOVA using the MIXED procedure with the REPEATED statement (SAS v. 9.4; SAS Institute Inc., Cary, NC). The time point number was the repeated variable, and cow was the subject of repeated measures.

For experiment 1, we present only the results for milking time points, because milking interval and milking time points did not interact (*P* > 0.1 for all variables). Fat content increased throughout milking, as did MFG diameter (d_4,3_) (*P* < 0.001; Table 1). In contrast, FFA concentration at t = 24 h decreased throughout milking (*P* < 0.001). Protein and lactose contents evolved in a similar manner: after peaking at time point 2, they decreased until the end of milking. The percentages of SFA and MUFA did not change during milking. Only the percentage of PUFA tended to increase (*P* = 0.090) and that of odd-chain FA increased after the first minute of milking (*P* = 0.015). The percentage of C16:0 increased during milking (*P* < 0.001), and the C18:1 *cis*-9/C16:0 ratio decreased after point 3 (*P* < 0.001). The percentages of C18:1 *trans*-11, C18:1 *trans*-10, and C18:1 *trans*-11/*cis*-7 (isomers indistinguishable by measurement) decreased during milking (*P* = 0.002, *P* < 0.001, *P* < 0.001, respectively; supplemental data: https://doi.org/10.15454/QTKBMA).

**Table 1:** Evolution of dairy parameters during milking in experiment 1 (Dutreuil et al., 2016)

Item	Portion of milking duration^1^							RMSE^2^	*P*-value
	1	2	3	4	5	6	7		
Milk yield, kg	2.32^c^	3.08^a^	3.23^a^	2.83^b^	1.76^d^	1.08^e^	0.30^f^	0.155	<0.001
Fat content, %	1.50^f^	2.36^e^	2.93^d^	4.69^c^	6.31^b^	7.16^a^	—3	0.925	<0.001
Protein content, %	3.29^b^	3.39^a^	3.36^a^	3.31^b^	3.17^c^	3.11^c^	—	0.100	<0.001
Lactose content, %	4.96^b^	5.23^a^	5.18^a^	5.02^b^	4.80^c^	4.78^c^	—	0.148	<0.001
MFG diameter (d_4,3_),^4^ µm	4.77^d^	5.17^c^	5.31^c^	5.73^b^	5.93^b^	6.15^a^	6.28^a^	0.114	<0.001
FFA^5^ t = 24 h, mEq/100 g of fat	0.76^a^	0.49^b^	0.45^b^	0.31^c^	0.35^bc^	0.40^bc^	—	0.084	<0.001
SFA,^6^ %	72.4	73.6	74.0	74.1	74.3	74.6	74.9	1.16	0.244
MUFA, %	20.6	21.0	21.1	21.1	21.0	20.7	20.4	0.52	0.535
PUFA, %	3.24	3.48	3.42	3.42	3.42	3.39	3.37	0.089	0.090
Odd-chain fatty acids,^7^ %	2.71^b^	2.73^b^	2.76^bc^	2.81^ab^	2.86^a^	2.86^a^	2.85^ac^	0.063	0.015
C16:0, %	32.6^d^	32.6^d^	33.5^cd^	34.2^c^	34.8^bc^	35.6^ab^	36.0^a^	0.68	<0.001
C18:1 *cis*-9/C16:0 ratio	0.416^ab^	0.429^a^	0.423^ab^	0.413abc	0.404^bc^	0.391^c^	0.379^d^	0.0141	<0.001

a–f Within a row, means with different letters differ significantly (*P* < 0.05).

1 The first 3 points correspond to the first 3 min of milking, respectively, and the last 3 points correspond to the last 3 min of milking, respectively. The middle point on the scale (point 4) corresponds to the mean value of samples from the intermediate minutes.

2 Root mean square error.

3 The volume of milk collected in the samplers was too low at the end of milking (point 7) to perform this analysis.

4 Milk fat globule (MFG) diameter: d_4,3_ = Σ(*N_i_* × *d_i_*^4^)/Σ(*N_i_* × *d_i_*^3^), where *N_i_*= number of MFG with diameter *d_i_*.

5 Free fatty acids measured after 24 h of storage at 4°C.

6 Sum of fatty acids from C4:0 to C24:0.

7 Sum of fatty acids from C5:0 to C19:0.

In experiment 2, fat content also increased throughout milking (*P* < 0.001; Table 2). The MFG diameter (*d_4,3_*) increased at the beginning of milking and quickly reached a maximum at time point 2 (*P* < 0.001). The concentration of FFA measured at t = 0 h decreased during the first 3 min and then reached a minimum (*P* < 0.001). The concentration of FFA measured at t = 24 h evolved in the same manner (*P* < 0.001), with extreme variability among cows at the end of milking (results not shown). Lipolysis did not change significantly. Lactose and protein contents decreased little but still significantly during milking (*P* < 0.001). Calcium content decreased throughout milking (*P* = 0.049). The percentage of SFA decreased at the beginning of milking and increased from point 4 until the end of milking (*P* = 0.001), whereas that of MUFA logically evolved in the opposite direction (*P* = 0.005; Table 2). The percentages of PUFA and odd-chain FA did not change. The percentage of C16:0 decreased at min 2 and then increased until the end of milking (*P* < 0.001), whereas the C18:1 *cis*-9/C16:0 ratio followed the opposite trend (*P* < 0.001). The percentages of C18:1 *trans*-9, and C18:1 *trans*-11/*cis*-7 decreased during milking (*P* < 0.001) (supplemental data: https://doi.org/10.15454/QTKBMA).

**Table 2:** Evolution of dairy parameters during milking in experiment 2 (Herve et al., 2017)

Item	Portion of milking duration^1^							RMSE^2^	*P*-value
	1	2	3	4	5	6	7		
Milk yield, kg	2.86^ab^	3.60^a^	3.92^a^	3.72^a^	2.62^ab^	1.64^b^	0.79^c^	0.468	<0.001
Fat content, %	0.69^e^	1.31^d^	2.16^d^	3.08^c^	4.48^b^	5.52^a^	6.26^a^	1.290	<0.001
Protein content, %	2.81^a^	2.75^b^	2.74^b^	2.72^bc^	2.68^bc^	2.66^bc^	2.64^c^	0.243	<0.001
Lactose content, %	5.18^ab^	5.22^a^	5.16^b^	5.08^c^	4.94^d^	4.86^e^	4.77^f^	0.077	<0.001
Calcium content, mg/kg	1,205^a^	1,191^ab^	1,188^b^	1,187^b^	1,171^b^	1,154^c^	1,147^c^	4.24	0.049
MFG diameter (d_4,3_),^3^ µm	3.57^b^	3.81^a^	3.93^a^	3.98^a^	4.02^a^	4.00^a^	4.00^a^	0.179	<0.001
FFA^4^ t = 0, mEq/100 g of fat	0.84^a^	0.47^b^	0.27^c^	0.20^c^	0.17^c^	0.17^c^	0.26^bc^	0.217	<0.001
FFA t = 24 h, mEq/ 100 g of fat	1.35^a^	0.87^b^	0.61^c^	0.53^c^	0.50^c^	0.62^c^	0.82^b^	0.145	<0.001
Lipolysis, mEq/100 g of fat	0.51	0.40	0.34	0.33	0.33	0.45	0.56	0.105	0.235
SFA,^5^ %	70.3^ab^	68.9^c^	69.1^c^	69.6^b^	70.5^a^	70.7^a^	70.9^a^	0.35	<0.001
MUFA, %	25.6^bc^	26.3^a^	26.1^ab^	25.6^ab^	24.8^c^	24.8^c^	24.6^c^	0.26	<0.001
PUFA, %	2.60	2.83	2.76	2.77	2.70	2.64	2.65	0.083	0.100
Odd-chain fatty acids,^6^ %	1.88^a^	1.88^a^	1.84^a^	1.81^ab^	1.79^ab^	1.75^b^	1.76^b^	0.032	0.004
C16:0, %	34.5^ab^	32.5^c^	32.8^c^	33.7^b^	34.8^a^	35.3^a^	35.8^a^	0.39	<0.001
C18:1 *cis*-9/C16:0 ratio	0.533^bc^	0.591^a^	0.581^a^	0.556^b^	0.516^c^	0.513^c^	0.502^c^	0.084	<0.001

a–f Within a row, means with different letters differ significantly (*P* < 0.05).

1 The first 3 points corresponded to the first 3 min of milking, respectively, and the last 3 points corresponded to the last 3 min of milking, respectively. The middle point on the scale (point 4) corresponded to the mean value of samples from the intermediate minutes.

2 Root mean square error.

3 Milk fat globule (MFG) diameter: d_4,3_ = Σ(*N_i_* × *d_i_*^4^)/Σ(*N_i_* × *d_i_*^3^), where *N_i_*= number of MFG with diameter *d_i_*.

4 Free fatty acids measured after 0 or 24 h of storage at 4°C.

5 Sum of fatty acids from C4:0 to C24:0.

6 Sum of fatty acids from C5:0 to C19:0.

Results from experiments 1 and 2 showed very similar trends for all traits except lactose content and C16:0. Lactose content differed between experiments at timepoints 1 and 5, and C16:0 differed only at timepoint 1, without biological or technical explanation. Milk fat content increased throughout milking in both experiments, which was expected. Ontsouka et al. (2003) and Rico et al. (2014) also observed this increase, which is due to the mechanisms of milk ejection and the physiology of the udder (presence of a cistern). Milk removed at the beginning of milking, corresponding to the cisternal milk, is less rich in milk fat than that removed at the end of milking, corresponding to the alveolar milk (Lollivier et al., 2002). Fat is secreted into the milk inside the lumen of the alveoli, and MFG are ejected into the udder cistern only when the myoepithelial cells contract. According to Ayadi et al. (2003), 89% of the total fat yield resides in the alveolar compartment and is only obtainable by contraction of the mammary gland. This contraction is stimulated by the action of oxytocin, which is not effective at the beginning of milking (Bruckmaier, 2001) and is released into the blood after 4 min of milking (Herve et al., 2016). Our results showed that mechanisms of ejection of milk fat during milking influenced the diameter of MFG. In both experiments, the diameter was smallest at the beginning of milking and increased until the end of milking. Our results are different from those of Rico et al. (2014), who showed that MFG size and distribution of MFG populations by size did not change during milking. In our experiment, only the smallest MFG were present in cisternal milk at the beginning of milking. The low fat content in cisternal milk may be due to the rising of MFG, which move in a direction opposite to the downward draining and newly secreted milk (Waldmann et al., 1999; Stelwagen, 2001). Another hypothesis is that only the smallest MFG can be released into the milk during the milking interval, when the myoepithelial cells do not contract. Large MFG should be released into the milk during contraction of the myoepithelial cells or ejected as oxytocin stimulates the myoepithelial cells as milking progresses. The concentration of FFA decreased during milking in both experiments, although lipolysis did not change in experiment 2. However, the high fat content at the end of milking could have rendered the MFG more sensitive to lipolysis, especially because this fat consisted of large MFG, which are more sensitive to lipolysis (Wiking et al., 2003). The presence and activity of LPL strongly influence the variation in lipolysis in milk. Milk LPL was not measured in these studies, but LPL was likely more present at the beginning of milking. The fact that LPL is found only in the nonfat fraction of milk (Chilliard, 1982) seems to support these hypotheses. At the beginning of milking, milk may have contained more LPL, which resulted in the high FFA concentration. This hypothesis requires verification because no data were found in the literature to explain the evolution of FFA or LPL during milking. The initially high FFA concentration could also have resulted from incomplete lipogenesis of triglycerides in the mammary epithelial cells (Chilliard, 1982) or secretion by the mammary epithelial cells into milk via simple diffusion (Schulz, 1991). Protein and lactose contents evolved throughout milking, which is consistent with results of Nielsen et al. (2005) and Sarikaya et al. (2005), who observed constant protein and lactose contents at the beginning of milking (up to 30% of milking duration) that then decreased until the end (from 3.3 to 3.0% and from 4.8 to 4.5%, respectively). Some milk FA varied throughout milking in both experiments, which could be related to MFG diameter (Lopez et al., 2011). In experiment 2, the evolution of calcium content during milking was related to that of protein content, because colloidal calcium is found in casein micelles (Neville and Peaker, 1979).

Both studies showed changes in milk composition throughout milking, especially the increase in MFG diameter and decrease in FFA concentration. It is also important to emphasize the high variability among cows related to duration of milking. The use of 2 different measuring devices for MFG diameter resulted in bias between the 2 experiments. The same parameter was measured, but considering the mode of operation of the Coulter Multisizer II system (distribution profile in the form of a frequency histogram divided into 106 classes of diameters between 1.42 and 20.01 μm), a mathematical model more precisely describing the distribution of the MFG population was created and could have overestimated the MFG diameter. In conclusion, milk fat composition and structure changed greatly during milking. Observing these dynamics in more dairy cows, along with analysis of other milk variables (e.g., enzymes), could provide more information about the evolution of milk fat composition and the lipolysis system during milking.
